# Characterisation of Translation Elongation Factor eEF1B Subunit Expression in Mammalian Cells and Tissues and Co-Localisation with eEF1A2

**DOI:** 10.1371/journal.pone.0114117

**Published:** 2014-12-01

**Authors:** Yuan Cao, Miriam Portela, Justyna Janikiewicz, Jennifer Doig, Catherine M. Abbott

**Affiliations:** Centre for Genomic and Experimental Medicine, University of Edinburgh, Institute of Genetics and Molecular Medicine, Western General Hospital, Edinburgh, United Kingdom; Oak Ridge National Laboratory, United States of America

## Abstract

Translation elongation is the stage of protein synthesis in which the translation factor eEF1A plays a pivotal role that is dependent on GTP exchange. In vertebrates, eEF1A can exist as two separately encoded tissue-specific isoforms, eEF1A1, which is almost ubiquitously expressed, and eEF1A2, which is confined to neurons and muscle. The GTP exchange factor for eEF1A1 is a complex called eEF1B made up of subunits eEF1Bα, eEF1Bδ and eEF1Bγ. Previous studies have cast doubt on the ability of eEF1B to interact with eEF1A2, suggesting that this isoform might use a different GTP exchange factor. We show that eEF1B subunits are all widely expressed to varying degrees in different cell lines and tissues, and at different stages of development. We show that ablation of any of the subunits in human cell lines has a small but significant impact on cell viability and cycling. Finally, we show that both eEF1A1 and eEF1A2 colocalise with all eEF1B subunits, in such close proximity that they are highly likely to be in a complex.

## Introduction

Translation elongation is mediated by a range of factors that are highly conserved throughout evolution and that are generally ubiquitously expressed. Translation elongation factor eEF1A delivers the aminoacylated tRNA to the ribosome; this is a GTP dependent process that is stimulated by a macromolecular complex called eEF1B. In lower eukaryotes eEF1B contains a guanine nucleotide exchange subunit eEF1Bα and a structural subunit eEF1Bγ, while higher eukaryotic cells have another guanine nucleotide exchange subunit eEF1Bδ (we are using the nomenclature proposed by Le Sourd et al ([1]).

eEF1Bα is the smallest subunit of the eEF1B complex and has guanine nucleotide exchange (GEF) activity. The C-terminal domain is considered to be necessary and sufficient for its GEF activity [2], and responsible for the interaction between eEF1Bα and eEF1A, while the N-terminal domain is involved in binding to the N-terminal domain of eEF1Bγ [3]. eEF1Bα has been found essential for cell growth in yeast [4], and mutation of this subunit enhances translation fidelity concomitant with a lower translational efficiency [5]. It is assumed that eEF1Bα promotes nucleotide exchange in eEF1A by disrupting interactions between GDP with the P-loop and switch regions of eEF1A [6].

eEF1Bδ is the metazoan-specific subunit of eEF1B; the C-terminus of eEF1Bδ is homologous with eEF1Bα [7] and contains the domain necessary for nucleotide exchange activity. The N-terminal domain of eEF1Bδ has a leucine zipper motif [8], indicating possible binding of other proteins, but this motif is not involved in the polymerization of eEF1Bδ monomers [9], and the N-terminal domain is not sufficient for the dimerization of eEF1Bδ [10]. eEF1Bδ has been found to exist as different isoforms resulting from alternative splicing, producing protein of around 35 kD. Recent studies have identified another eEF1Bδ protein isoform of around 70–80 kD, termed eEF1BδL. The mRNA encoding eEF1BδL contains an extra exon, exon 3, which is skipped in the mRNA transcripts of other isoforms and is tissue specific, expressed only in brain, spinal cord and testis. This exon encodes a 367-amino-acid long N-terminus, which contains a putative nuclear localization signal at amino acids 86–93 [11]. The resulting isoform is expressed in the nucleus where it participates in the heat shock and stress response [11].

eEF1Bγ is the eukaryotic specific subunit of eEF1B. The N-terminal domain of eEF1Bγ contains a region of homology to the theta class of glutathione S-transferases (GSTs) [12]. The role of eEF1Bγ in translation elongation is not well understood. eEF1Bγ is usually found tightly associated with eEF1Bα and can be isolated from eEF1Bα only under strong denaturing conditions. Research using *Artemia* showed that the nucleotide exchange rate of eEF1Bα is higher in the presence of eEF1Bγ. eEF1Bγ is also likely to be involved in directing other subunits in the eEF1B complex [13] and to play a role in scaffolding for the eEF1B complex [1] as it is highly associated with membrane and cytoskeleton structures.

Although the components of eEF1B have been reasonably well characterised, and eEF1B is considered to form a reversible macro complex with eEF1A (eEF1H) to mediate the guanine nucleotide exchange on eEF1A, how the three subunits of eEF1B combine and interact with eEF1A remains unclear and there is inconsistency between the models proposed. The first structural model proposed was based on *in vitro* reconstitution experiments using different combinations of the subunits purified from rabbit liver, as well as published information about eEF1H subunits from *Artemia* by other groups [14]. They suggested a protomer composed of valyl-tRNA and eEF1H, which were associated through eEF1Bδ. Two such protomers could bind to each other via the leucine zipper motif on the N-terminus of two eEF1Bδ subunits. A subsequent study of *Artemia* suggested a different structural model wherein eEF1Bγ binds to both eEF1Bα and eEF1Bδ, each of which binds to a eEF1A subunit [15], and further models with different features were proposed by other groups [10],[16],[17],[18].

Although the above models are different from each other, some consistent features emerge. Firstly, it is believed that eEF1Bα and eEF1Bγ are tightly associated and can only be separated under denaturing conditions [19]. Secondly, eEF1Bα and eEF1Bδ show no affinity for each other. Finally, the binding sites of eEF1Bα and eEF1Bδ to eEF1Bγ locate on the N-terminus of the three proteins, while the C-terminus of eEF1Bα and eEF1Bδ harbors the binding sites for eEF1A.

A further complication arises from the fact that eEF1A is found as two isoforms in vertebrates, each encoded by a separate gene and each expressed in different cell types. Whilst eEF1A1 is almost ubiquitously expressed, it is downregulated postnatally in neurons and muscle and replaced by eEF1A2. One of the studies that took eEF1A2 into account was a series of yeast two-hybrid (Y2H) analyses, where the cDNAs of both isoforms of eEF1A and all three eEF1B subunits, were cloned into Y2H expression vectors respectively to map the interaction patterns between the proteins. It was found that in contrast with eEF1A1, eEF1A2 has little or no affinity for eEF1Bα and eEF1Bδ [10], the two eEF1B subunits that have GTP exchange activity. This finding was surprising as the two isoforms of eEF1A would be predicted to have similar abilities to bind to eEF1B, particularly eEF1Bα. The amino acid sequences of the two isoforms of human eEF1A are 92% identical. Comparative three-dimensional models of human eEF1A1 and eEF1A2 on the basis of the crystal structure of homologous eEF1A from yeast show that almost all the residues that differ between eEF1A1 and eEF1A2 are found on one side of the molecule, while the binding sites for eEF1Bα are on the other side [20]. Furthermore, whereas eEF1A1 which binds GTP more strongly than GDP, eEF1A2 shows more affinity for GDP than GTP [21], suggesting that eEF1A2 might be more dependent than eEF1A1 on the presence of a GTP exchange factor. One explanation proposed for the different affinities of the two isoforms of eEF1A to eEF1B was the potential existence of a different GEF for eEF1A2 [10]. Multiple chromosomal isoforms of human eEF1Bα have been identified, one of which transcribes a brain- and muscle-specific cDNA [22]. This expression pattern is in accordance with that of eEF1A2 and was suggested to act as the GEF specifically for eEF1A2. However, as this gene is intronless and absent from mice this seems unlikely to be the explanation [23].

In order to resolve this apparent contradiction, we used proximity ligation assays (PLA) to ask whether both eEF1A2 and eEF1A1 can bind to eEF1B subunits. We also analysed expression and the effects of ablating expression of eEF1B subunits in a range of cell lines and tissues.

## Results

### Expression analysis in cell lines and tissues

**Figure 1:**
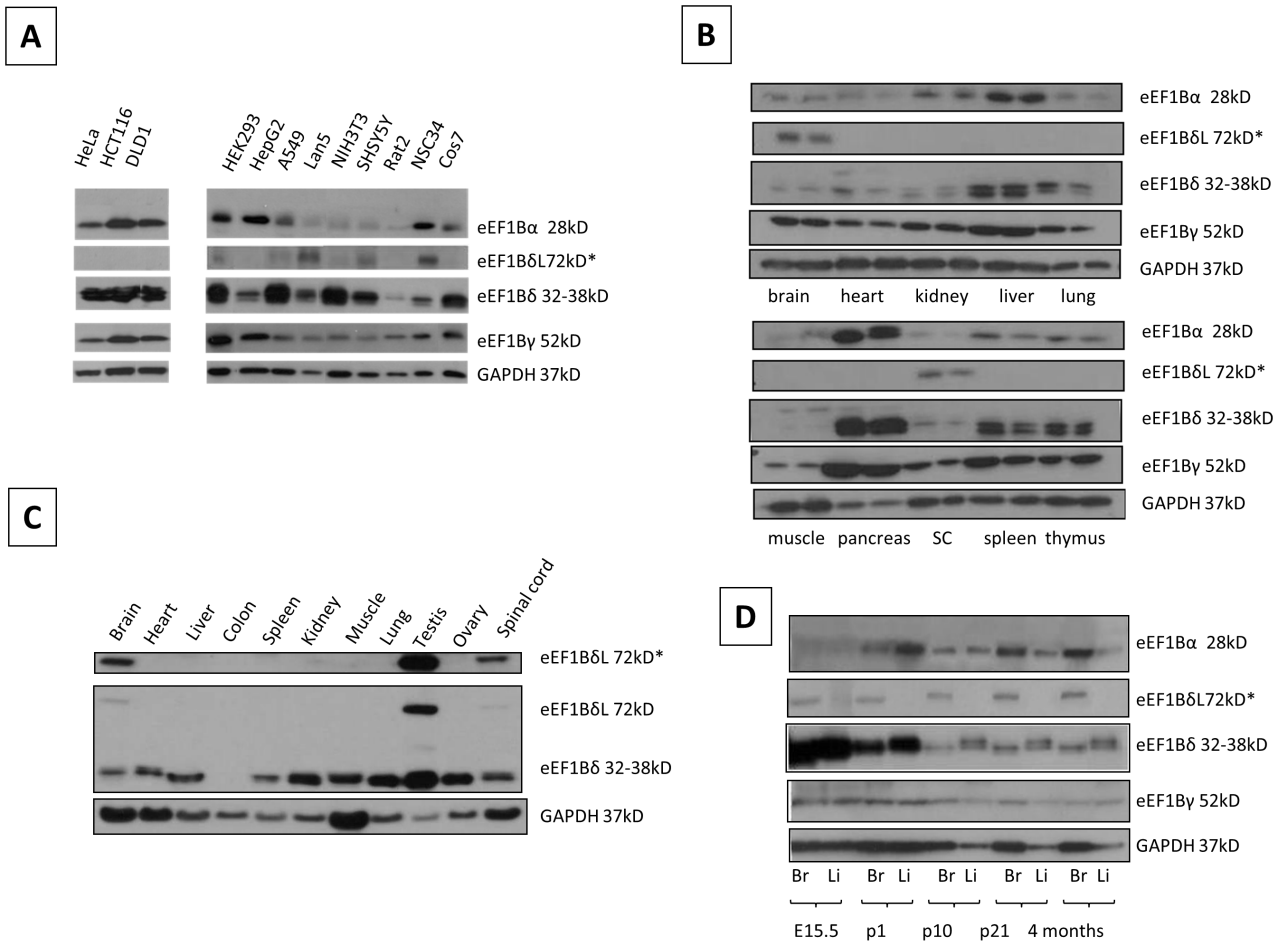
Expression analysis of eEF1B subunits in cell lines and tissues. Panel A: Immunoblot of eEF1Bα, eEF1Bδ and eEF1Bγ protein expression in cell lines. GAPDH was used as a loading control. Panel B: Immunoblot of eEF1B subunits in 24-day old mice. Each tissue is shown twice, the left hand sample is from a wild type mouse and the right hand sample from a wasted homozygous mouse. SC =  spinal cord. The second row shows a longer exposure of the eEF1Bδ blot, revealing the expression of the longer isoform. GAPDH was used as a loading control. Panel C: Immunoblot of eEF1Bδ in an extended panel of mouse tissues showing brain and testis-expression of the longer isoform (top panel shows a longer exposure). GAPDH was used as a loading control. Panel D: Immunoblot of eEF1B subunits expression in brain (Br) and liver (Li) throughout late embryonic and postnatal mouse development. GAPDH was used as a loading control.

Initially we compared expression of each of the three subunits of eEF1B in a panel of cell lines using Western blots. Twelve cell lines were examined, three of which are untransformed (NIH3T3, Rat2 and HEK293). eEF1Bα was detected in all cell lines but was present at lower levels in the NIH3T3, SHSY5Y, Rat2 and NSC34 cells, all of which are untransformed or neuronal in origin. eEF1Bδ at ∼35 kD was present in all cells but at a low level in Rat2 cells. The other splice form of eEF1Bδ that includes exon 3, at 72 kD (labelled eEF1BδL) was only apparent after long exposures in certain cell lines, notably NSC34, a spinal cord by neuroblastoma fusion line with a neuronal phenotype. The expression pattern of eEF1Bγ in all cell lines tested were similar, with no obvious differences apart from strong expression in HEK293 cells (Figure 1A). In general, all subunits are expressed in the majority of cell lines.

We then went on to look at expression in a range of mouse tissues. Panel B of Figure 1 shows expression in paired samples from wild-type and wasted (eEF1A2-null) mice at 24 days of age. The lack of eEF1A2 has no effect on expression, but it can be seen that expression varies between tissues. Every tissue tested expresses all eEF1B subunits, with particularly high expression seen in pancreas. eEF1Bα is expressed at a low level in brain, spinal cord, heart, lung and muscle. These results are generally consistent with those in RNA expression databases such as those in GEO profiles, which also show widespread expression of all subunits at the RNA level.

eEF1Bδ shows different variants, with the 72 kD form expressed only in brain and spinal cord. Other isoforms cluster around 32 to 38 kD and are most strongly expressed in liver, pancreas, spleen and thymus. Panel C shows expression of the eEF1Bδ isoforms in more detail including a longer exposure of the Western, confirming strong expression of the eEF1BδL form at the protein level in brain, spinal cord and testis.

eEF1Bγ is expressed at a similar level in all the tissues tested, except for muscle, which shows weak expression, and pancreas, where it is highly expressed, as for the other subunits (panel B).

Panel D of Figure 1 shows Western blots for each subunit each in brain and liver from mice at different developmental stages. It can be seen that whereas expression of eEF1Bα is barely detectable before birth, eEF1Bδ is expressed at much higher levels at E15.5 and P2 than at later stages, in both liver and brain. eEF1Bγ shows a similar but less dramatic trend. The eEF1BδL isoform, in contrast, is expressed in brain at all ages but showing a slight increase with age.

**Figure 2:**
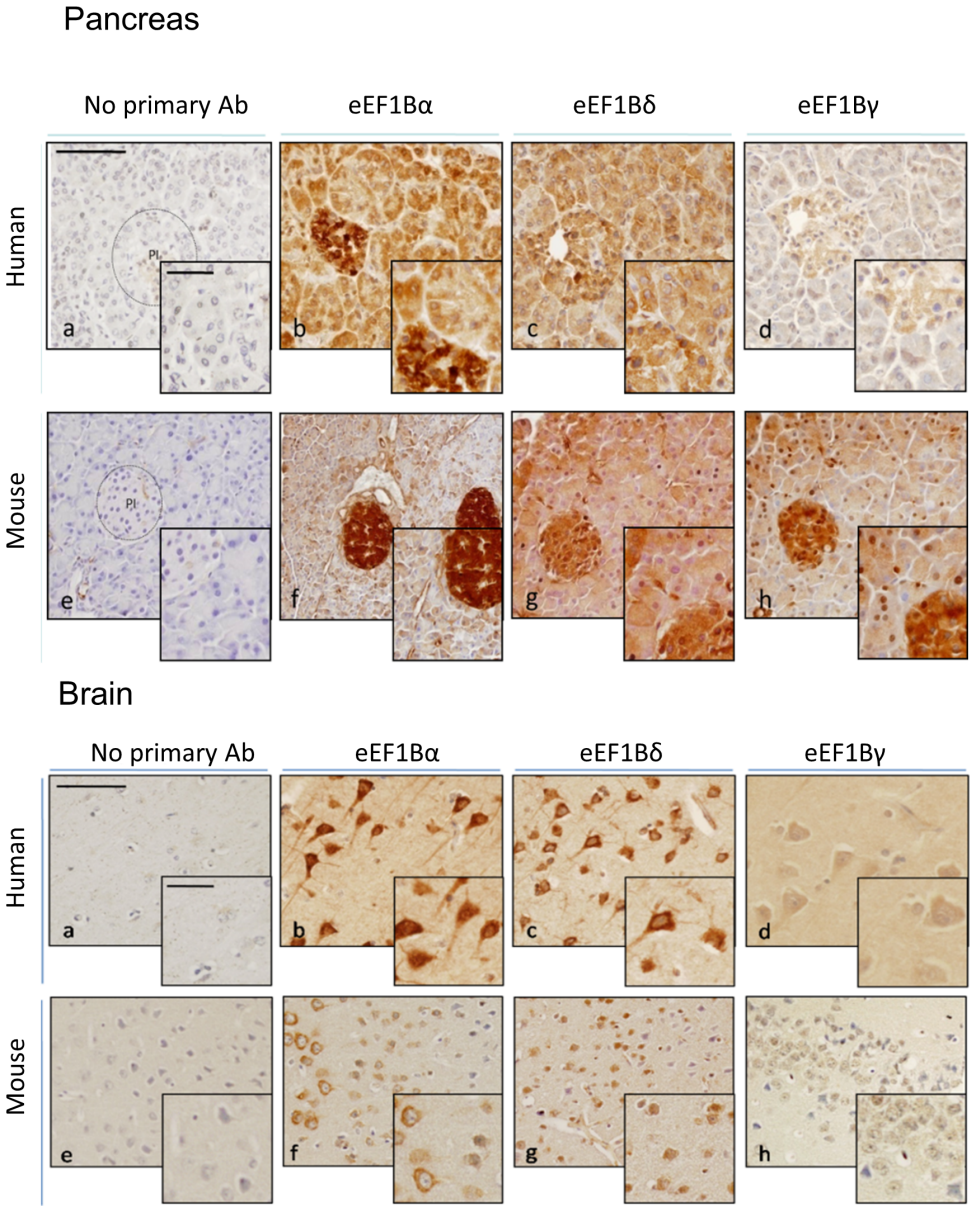
Immunohistochemistry of eEF1B subunits in human and mouse tissues. Immunohistochemistry of eEF1B subunits in human and mouse brain and pancreas. Proteins detected through primary antibody incubation, HRP mouse + rabbit secondary antibody and subsequent incubation with DAB. Positive signal is indicated by the presence of brown DAB reaction product. Panels show eEF1Bα (**b** and **f**), eEF1Bδ(**c** and **g**) and eEF1Bγ (**d** and **h**) Incubation with secondary antibody only was used as negative control (**a** and **e**). Bar (top left micrograph) represents 100 and 50 µm respectively.

We then used immunohistochemistry to compare expression of the subunits in a selection of human and mouse tissues. Figure 2 shows the results obtained in pancreas andbrain. Staining in the pancreas shows similarly high levels of expression of each subunit in both human and mouse, with particularly strong expression throughout the islets. Both eEF1Bα and eEF1Bδ are strongly expressed in neurons, although in the case of eEF1Bδ we are unable to tell whether this is staining is specific for the long isoform, as there are no antibodies that distinguish the two. However, there is little sign of nuclear expression, the reported site of eEF1BδL in cells [11].

### Ablation of eEF1B subunits in mammalian cells

**Figure 3:**
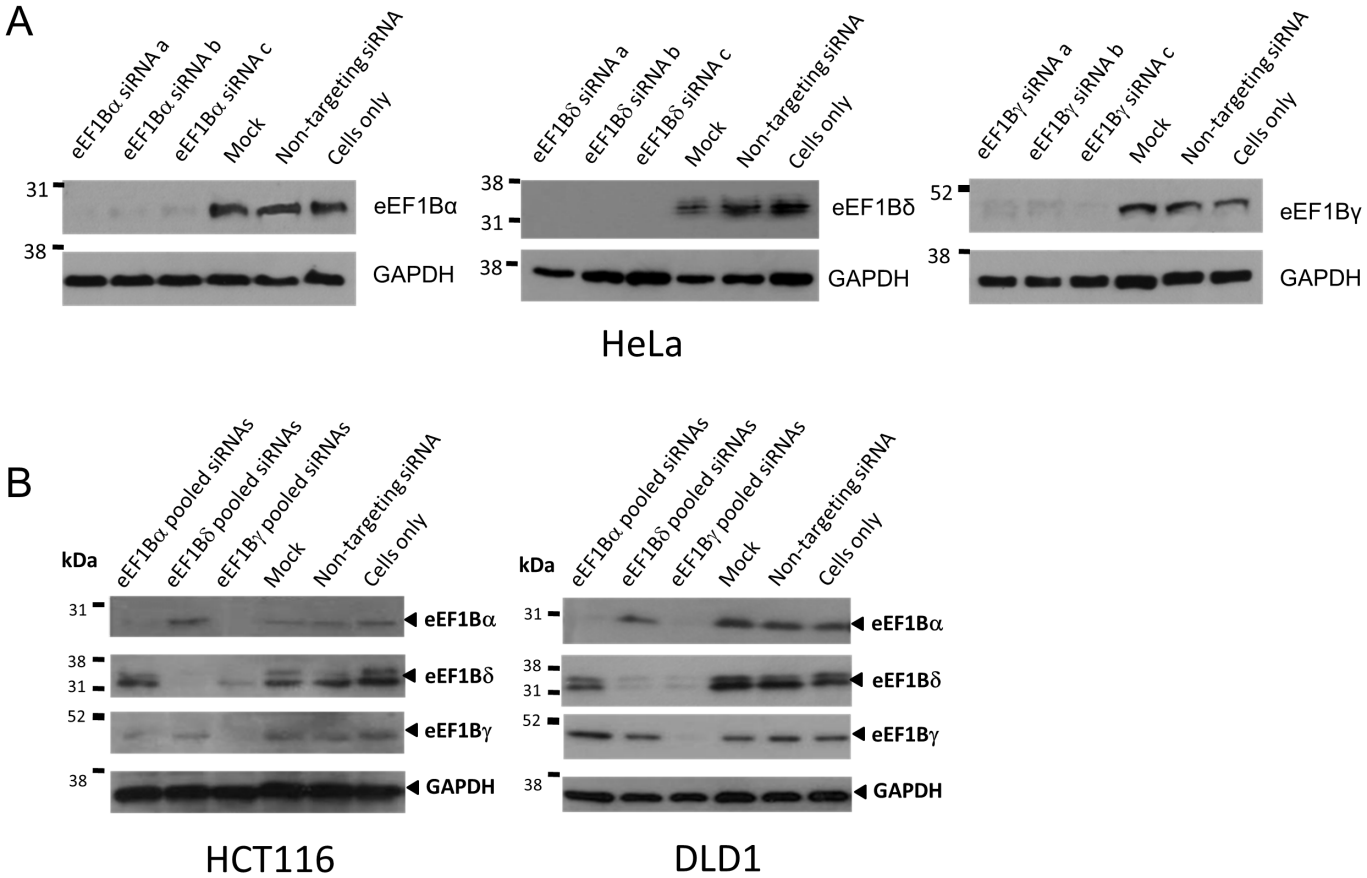
Using RNA interference to knock down expression of eEF1B subunits. Immunoblots of protein extracts from cell lines after RNA interference. Panel A: eEF1Bα, eEF1Bδ and eEF1Bγ protein level efficiently knocked down by three different siRNAs in HeLa cells 72 h after transfection. GAPDH was used as a loading control. Panel B: eEF1Bα, eEF1Bδ and eEF1Bγ protein level efficiently knocked down by three different siRNAs in HCT116 and DLD1 cells 72 h after transfection. GAPDH was used as a loading control.

The protein expression of each eEF1B subunit and GAPDH from cells transiently transfected with three different siRNAs for each of the eEF1B subunits and a scrambled siRNA were compared by Western blot. All three siRNAs targeting eEF1Bα, eEF1Bδ and eEF1Bγ substantially reduced the respective protein at 72 hours in comparison with cells treated with scrambled siRNA (Figure 3a).

These experiments were then repeated in two further cell lines, HCT116 and DLD1 cells (Figure 3b). We also examined whether the ablation of any one subunit affected the expression of the other subunits in these cell lines. The results can be seen in Figure 3b. This showed that siRNAs targeting eEF1Bα consistently ablated expression of the target protein but also showed some variable and inconsistent down-regulation of the other two subunits. Targeting of eEF1Bδ was efficient in each cell line, and had no observable effect on either of the other two subunits. In contrast, ablation of eEF1Bγ consistently resulted in downregulation of both eEF1Bα and eEF1Bδ in all cell lines tested, possibly due to a reduction in stability of the complex.

**Figure 4:**
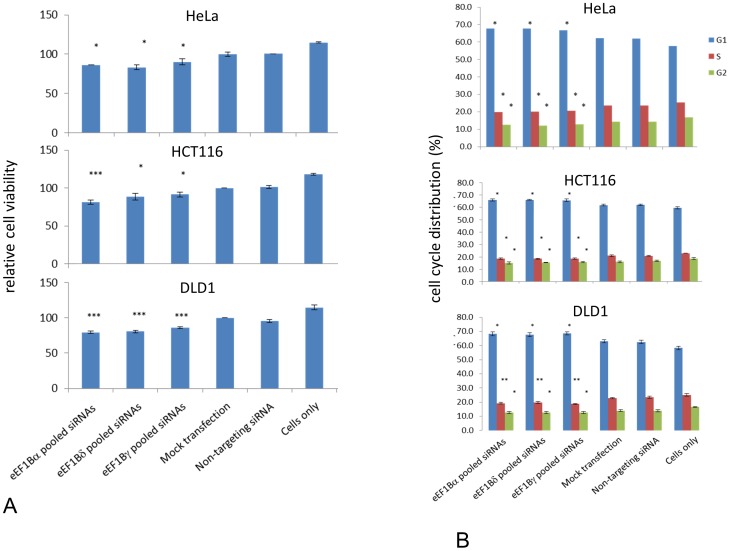
Viability and cell cycle distribution of cells after ablation of eEF1B subunits. Panel A: A decrease in cellular metabolism is observed when any of the eEF1B subunit protein level is decreased by siRNAs in HeLa, HTC116 and DLD1 cells. Cell metabolism was assessed by the Alamar blue assay. Data were obtained from the mean of three or more independent experiments in HeLa, HCT116 and DLD1 cells, with more than 10 wells each. Error bars indicate +- SEM; n of wells>10; n = 3−4; *, P<0.05; ***, P<0.001 from non-targeting siRNA. Panel B: Knockdown of eEF1B subunits leads to altered cell cycle profile in three cell lines: representative images of the flow cytometry analysis. Error bars indicate +- SEM; n = 3; *, P<0.05; **, P<0.01 of non-targeting siRNA.

We then examined the effect of downregulation of eEF1B by RNAi on cell viability using Alamar Blue. The mean of at least three independent experiments was plotted relative to the viability of mock transfected cells. Significance was assessed using t-tests to compare results from cells transfected with eEF1B subunits targeting siRNAs compared to non-targeting siRNA. Knockdown of each eEF1B subunit slightly reduced cell viability, by around 14% (eEF1Bα by 14%, eEF1Bδ by 17% and eEF1Bγ by 10%, when compared with cells transfected with a scrambled siRNA (Figure 4a; p<0.05). In HCT116 cells, eEF1Bα, eEF1Bδ and eEF1Bγ siRNA induced knockdown resulted in a reduction of cell metabolism of over 19% (p<0.001), 12% (p<0.05) and 10% (p<0.05) respectively compared with cells transfected with non-targeting siRNAs). In DLD1 cells this effect was even more pronounced, with a reduction in cell viability of at least 20% when either eEF1Bα or eEF1Bδwas downregulated, and 14% for eEF1Bγ compared to cells transfected with a non-targeting siRNA. As the downregulation of some subunits, notably eEF1Bγ, can affect the expression of the other subunits, it is difficult to impart any specificity to these results, but it is clear that loss of eEF1B has an effect on viability/cell metabolism in a variety of human cell lines.

Given the links between the cell cycle and eEF1B subunits and the reduction in viability observed when eEF1B subunits are depleted, we went on to examine the cell cycle profile of cells in which eEF1B subunits had been downregulated by RNAi. Cells were collected 72 hours after transfection, stained with propidium iodine and analysed by flow cytometry. Figure 4b shows graphs representing the mean of three independent experiments in which depletion of eEF1B subunits was confirmed by Western blotting.

Depletion of eEF1B subunits by RNAi in HeLa cells increased the proportion of cells in G0/G1 phase by 7%, with a concomitant decrease in cells in S-phase by 13% and in G2/M phase by 10% when compared with cells transfected with a scrambled siRNA (p<0.05; Figure 4b). Untreated cells showed a reduction in the number of cells in G0/G1 and an increase in the S- and G2/M-phases compared with mock transfected cells, but no statistically significant differences were seen in the cell cycle distribution of cells transfected with scrambled siRNA and mock transfected cells. Ablation of any eEF1B subunit is therefore associated with a small but significant decrease in the proportion of cells in S and G2/M phases and an increase of cells in G0/G1 phase.

In HCT116 cells, down-regulation of eEF1B subunits increased the proportion of cells in G0/G1 by 6%, and reduced the proportion of cells in S-phase by 10% and in G2/M phase by 5% in comparison to cells with scrambled siRNA (p<0.05; Figure 4b). Similarly, in DLD1 cells, knocking down eEF1B subunits increased by 8% the proportion of cells in G0/G1 compared with cells transfected with non-targeting siRNA (p<0.05). Cells in S-phase decreased by over 15% (p<0.01) and cells in G2/M phase decreased by more than 9% (p<0.05; Figure 4b). In all cell lines studied therefore, ablation of eEF1B is associated with a small but significant decrease in the proportion of cells in S and G2/M phases and an increase of cells in G0/G1 phase.

#### eEF1B subunits co-localise with eEF1A2 using PLA

eEF1B subunits were found not to bind to eEF1A2 in a yeast 2 hybrid experiment [10]. This was unexpected given the position of the eEF1B binding site relative to amino acid differences between eEF1A1 and eEF1A2 [20]. We therefore sought to look in mammalian cells for evidence of interaction with eEF1A2.

**Figure 5:**
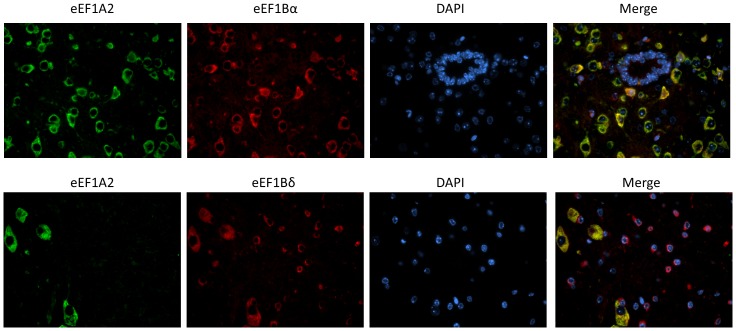
Expression in mouse spinal cord. IF images of the expression of eEF1A2 and eEF1Bα (top panel) or eEF1Bδ (bottom panel) on mouse spinal cord.

Initially, we used immunofluorescence on sections of mouse spinal cord to see if there was any evidence for co-localisation of eEF1A2 with eEF1Bα and eEF1Bδ (Figure 5). It can be seen that there is strong co-localisation of both eEF1B subunits with eEF1A2 in the neurons (we were unable to test eEF1Bγ because of issues with antibody specificity under these conditions). Both eEF1B subunits also showed expression in non-neuronal cells that do not express eEF1A2.

In order to obtain more direct evidence for binding, we used the proximity ligation assay (PLA). PLA is based on dual binding by a pair of probes to the two proteins of interest via two specific antibodies raised in different species, in order to generate DNA strands, which then are amplified and serve as surrogate markers for the detected protein molecules [24],[25]. PLA has the advantage of investigating endogenous protein interactions *in situ*, either in tissues or cultured cells directly.

**Figure 6:**
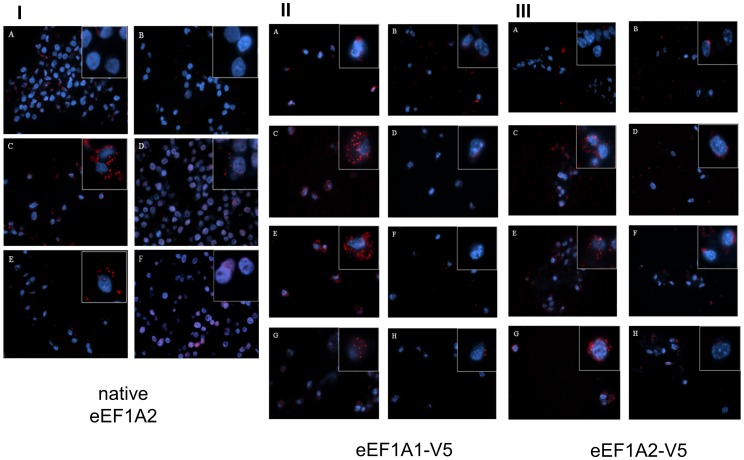
Proximity ligation assays for eEF1A and eEF1B. Panel I: PLA on HeLa cells. A. Negative control with both primary antibodies omitted. B. eEF1A2 antibody only. C. PLA of eEF1A2 and eEF1Bα. D. PLA of eEF1A2 and eEF1Bδ. E. PLA of eEF1A2 and eEF1Bγ. F. PLA of eEF1A2 and TK1 as negative control. Images in the squares are higher magnification of selected areas. Panel II: PLA on NIH-3T3 cells stably expressing V5-tagged eEF1A1. A. Negative control with both primary antibodies omitted. B. V5 antibody only. C. PLA of eEF1Bα and V5. D. PLA of eEF1Bα and TK1. E. PLA of eEF1Bδ and V5. F. PLA of eEF1Bδ and TK1. G. PLA of eEF1Bγ and V5. H. PLA of eEF1Bγ and TK1. Images in the squares are higher magnification of selected areas. Panel III: PLA on NIH-3T3 cells stably expressing V5-tagged eEF1A2. A. Negative control with both primary antibodies omitted. B. V5 antibody only. C. PLA of eEF1Bα and V5. D. PLA of eEF1Bα and TK1. E. PLA of eEF1Bδ and V5. F. PLA of eEF1Bδ and TK1. G. PLA of eEF1Bγ and V5. H. PLA of eEF1Bγ and TK1. Images in the squares are higher magnification of selected areas.

Figure 6A shows PLA performed in HeLa cells, which express both eEF1A1 and eEF1A2 [26]. Although in one report the longer form of eEF1Bδ was found in HeLa cells [11], the cells we used expressed only shorter eEF1Bδ isoforms (Figure 1A). In HeLa cells eEF1A2 gave positive PLA signals with all eEF1B subunits (Figure 6IC,D and E). PLA using no antibody (5IA), eEF1A2 antibody only (6IB) and eEF1A2 and TK1 antibodies (6IF, a further negative control) gave neither PLA signals nor any background, demonstrating that the signals produced by the eEF1A2/eEF1Bα, eEF1A2/eEF1Bδ and eEF1A2/eEF1Bγ antibody pairs are genuine and specific, and that eEF1A2 does co-localise with eEF1Bα and eEF1Bδ in HeLa cells at the resolution that can be detected by PLA.

In order to confirm these results and to show that under these conditions eEF1B subunits also interacted with eEF1A1 (as predicted from early eEF1 complex purification experiments) we used cell lines which stably express either eEF1A1 with a V5 tag or eEF1A2 with a V5 tag (J. Janikiewicz, unpublished). Our antibodies raised against eEF1A1 are not specific under these conditions, and also recognise eEF1A2, so the use of these cell lines permitted us unambiguously to distinguish between the two isoforms.

In V5-eEF1A1 expressing NIH-3T3 cells, a V5 antibody gave positive PLA signals with all eEF1B subunits as expected (Figure 6II). The signals for V5/eEF1Bγ (Figure 6IIG) were not as numerous as those for V5/eEF1Bα or V5/eEF1Bδ (Figure 6IIC and E), but were nevertheless considerably more numerous than those in any of the negative controls. In V5-eEF1A2 expressing NIH-3T3 cells similar results were observed (Figure 6III). The PLA signals were slightly less strong than in V5-eEF1A1 transgenic cells, but the combined results of this analysis and of endogenous eEF1A2 in HeLa cells strongly suggest that eEF1A2 is able to bind to a complex containing all three eEF1B subunits.

## Discussion

We have shown that although each of the components of the eEF1B complex is widely expressed, their expression is not uniform in either tissues or cell lines. eEF1Bδ, in particular, shows an additional layer of complication with alternative splicing and the use of a long additional 5′ exon that gives rise to a much larger protein isoform whose expression is confined to brain (this study, [11]) and testis [11]. In this case, the long isoform has a different subcellular location and function, as it localised to the nucleus where it is involved in induction of the heat shock response. With this exception, all the subunits do, however, appear to be ubiquitously expressed in cell lines and tissues. Both eEF1Bδ and, to a lesser extent, eEF1Bγ, are strongly expressed at E15.5 in both brain and liver, with expression being downregulated by p10. Interestingly, eEF1Bα and eEF1Bδ show an almost reciprocal expression trend during development, with eEF1Bα being expressed more strongly postnatally in both brain and liver, and eEF1Bδ being extremely highly expressed in both tissues at E15.5 and P1 but then dropping markedly by P10. In adult mice, pancreas and liver show consistently high levels of all subunits (Figure 1). Immunohistochemistry shows that the staining in pancreas is particularly intense in pancreatic islets, although this is less marked with the human sample tested (Figure 2). It is impossible to draw solid conclusions from a single sample, though. Both eEF1Bα and eEF1Bδ are strongly expressed in neurons, more so than in glia, and again this is seen in both mouse and human samples.

All the subunits proved eminently amenable to being knocked down using RNA interference (Figure 3), being reduced to near undetectable levels in HeLa cells at 72 hours; similar results were obtained with two other cell lines. Subsequent experiments showed that knocking down eEF1Bγ also affected expression of the other two subunits, so the effects of ablating this subunit cannot be judged in isolation. Knocking down expression of any one of the subunits, in each cell line tested, had a small but significant effect on cell viability, and concomitant modest alteration in cell cycle profile with a reduction of cells in S phase. Surprisingly, knocking down eEF1Bγ seemed to have the lowest effect on viability in two of the cell lines, even though knocking down this subunit also affects expression of eEF1Bα and eEF1Bδ. The difference, if real, might be attributable to the effects on viability being mediated by an imbalance in the subunits rather than a general decrease in expression of the whole complex. In contrast, eEF1Bα has been shown to be essential for growth in yeast [4]. It is possible that this would also be true of mammalian cells over an extended period of time, but certainly our results suggest that they are able to survive and grow over several days with only very low levels of eEF1B. This could imply that mammalian eEF1A isoforms are less dependent on eEF1B for their function in protein synthesis; yeast do not have eEF1A2, and it would be formally possible that the presence of eEF1A2 rescues the loss of eEF1B. However, the biochemical analysis of purified eEF1A2 would suggest that if anything it would be more dependent on GTP exchange than would eEF1A1 [21]. Moreover, the DLD1 cell line was selected for this experiment because, unlike HeLa and HCT116 cells, it does not express eEF1A2 (unpublished observations). The viability of this cell line was slightly lower after ablation of eEF1B than the other cell lines, but not to an extent that seems likely to be significant.

As an important part of the cellular machinery that regulates protein translation elongation as well as being involved in other cellular functions, the structure of eEF1H has been broadly studied. eEF1H is composed of eEF1A and eEF1B, and several models have been proposed for relative placement of each component. However, as there is much inconsistency among these models, further studies were required, particularly in regard to possible differential binding of eEF1A1 and eEF1A2 to eEF1B. In particular, although we showed that regions containing sequence differences between eEF1A1 and eEF1A2 proteins do not harbour eEF1B binding sites, suggesting the two isoforms have similar capability of binding to eEF1B [20], yeast 2 hybrid experiments had previously demonstrated interactions between eEF1A1 and eEF1B, but no interactions between eEF1A2 and eEF1B subunits. This study suggested that eEF1A2 might use a different GTP exchange factor [10]. We therefore used the *in situ* Proximity Ligation Assay (PLA) technique to attempt to resolve this discrepancy. PLA has been proved to be an efficient and straightforward method to examine endogenous protein-protein interactions *in situ*, avoiding the possible artefacts from experimenting with isolated or exogenous proteins.

As a first step, we carried out co-localisation analysis of eEF1A2 with eEF1Bα and eEF1Bδ on spinal cord sections and were able to demonstrate clear co-localisation in spinal cord motor neurons. PLA, however, gave false positive signals when used in fixed spinal cord in our hands, so we turned to cell lines. These false positives have also been reported in a study of fixed but not fresh mouse brain [27], possibly due to the deleterious effect of aldehydes on DNA that might benefit the binding of oligonucleotides to the probes during the PLA reaction [27]. These false positives were not observed using cultured cells fixed with the same methods, suggesting that neurons may be more sensitive to the effects of aldehydes on DNA.

PLA was then carried out using HeLa cells, which strongly express both eEF1A1 and eEF1A2. A clear PLA signal was obtained for each of the eEF1B subunits when used with an antibody against native eEF1A2; controls using no antibodies or an antibody to TK1 were all negative.

Since no specific anti-eEF1A1 antibody was available, the PLA of eEF1A1 with eEF1B was carried out on NIH-3T3 cell lines that stably expressed either V5-tagged eEF1A1 or V5-tagged eEF1A2. In both V5-tagged 1A1-3T3 cells and 1A2-3T3 cells, PLA of V5 and all three eEF1B subunits gave positive PLA signals whilst the negative controls were all weak or completely clear. As comparable results were obtained for both eEF1A isoforms, both eEF1A1 and eEF1A2 appear to bind to eEF1B. It seems possible, therefore, that the fusion protein expressed in yeast cells may have failed to keep the native conformation and consequently influenced protein interactions in the Y2H experiments [10]. It is also conceivable that differences in the ability of each isoform to self-associate could have affected the results [33]. With the same Y2H system eEF1A2 in fusion with GAL4 DNA-binding domain was competent in interaction with other eEF1A-binding proteins identified in an Y2H screening [10],[28], but perhaps the fusion protein was modified in a way that masked the binding sites for eEF1B.

The other major difference between the result in our study and the Y2H experiments is that in transgenic NIH-3T3 cells we also detected an interaction between V5-tagged eEF1A1 and eEF1Bγ, which was not found in the Y2H experiments. This is presumably because that eEF1A and eEF1Bγ are not directly binding each other, but form a complex bridged by another eEF1B subunit, as suggested in most of the eEF1H structure models proposed so far. In PLA the maximum distance between the two probes that allows DNA hybridization and thus PLA signal is around 16 nm [27]. Including the two primary antibodies and the two probes, the distance for two proteins to be recognized as being in proximity by PLA is estimated at roughly 30–50 nm, depending on the sizes of the antibodies used [29],[30]. The limitation of the PLA technique in terms of distinguishing between binding to specific subunits rather than proximity due to binding of the test protein to an entire complex means that we can not deduce unequivocally whether there are differences in the binding of eEF1A1 and eEF1A2 to specific subunits. We can, however, say that both eEF1A1 and eEF1A2 bind to the eEF1B complex.

In conclusion, we suggest that both eEF1A isoforms can bind to eEF1B subunits in vivo. This does not preclude the possibility of a further GTP exchange factor binding to eEF1A2, but this result coupled with our previous comparative homology modelling makes it likely that eEF1A2 does indeed use eEF1B as a GTP exchange factor. Furthermore, the STRING database of protein-protein interactions version 9.1 shows evidence for binding of eEF1Bα and eEF1Bδ to eEF1A2 in human cells, and of binding of eEF1Bδ and eEF1Bγ to eEF1A2 in mouse cells. Again, it is likely that these results indicate binding of the eEF1B complex to eEF1A2 rather than telling us about binding of individual subunits, as all available biochemical evidence suggests strongly that the gamma subunit does not bind directly to eEF1A. Mammalian cells appear to be able to survive for at least periods of a few days in culture in the absence of any given eEF1B subunit, but of course the absence of the whole complex could be lethal.

The recent discovery of missense mutations in eEF1A2 that cause epilepsy, severe intellectual disability and autism is likely to cause more focus on the role of eEF1B binding, as the G70S mutation abuts the eEF1B binding site[34],[35]. It is also of note that a homozygous splice site mutation in the gene encoding eEF1Bα has been found in a family with recessive intellectual disability, suggesting that translation elongation factors may have an important neurodevelopmental role to play in cognition [31]. Further studies on the role of eEF1B in neurons is now a priority.

## Methods

### Mice

Mice were housed in the Biomedical Research Facility (BRF) at the University of Edinburgh. All mice were maintained in accordance with Home Office regulations and all protocols had been approved by the local ethics committee of the University of Edinburgh. Wasted mice were closely observed for overall clinical condition and were euthanized where necessary to avoid suffering.

### Cell culture

HeLa, HCT116 and DLD1 cells were all obtained locally and cultured according to ATCC guidelines.

### Western blotting

Westerns were carried out using antibodies and techniques as previously described [32], with the following antibodies: anti-eEF1Bα from Proteintech (Manchester, UK; 1/2000) or Abcam (Cambridge, UK; 1/400), anti-eEF1Bδ from Proteintech Group (Manchester, UK; 1/3000), anti-eEF1Bγ from Abnova (Taipei, Taiwan; 1/2000) or Abcam (Cambridge, UK; 1/1000) and anti-GAPDH from Chemicon (Merck Millipore, Watford, UK; 1∶30,000). Calbiochem (Merck Millipore, Watford, UK) Rapid Step ECL was used for detection.

### Immunohistochemistry

Slides of human tissues were obtained from Biochain (AMS Biotechnology, Abingdon, UK). Paraffin embedded sections of human and mouse tissues were deparaffinised, blocked in peroxidise blocking solution for 5 minutes and then washed and blocked in goat serum diluted 1∶5 with PBS for 10 minutes. Primary antibody was added as follows: anti-eEF1Bα from Proteintech (Manchester, UK; 1/100), anti-eEF1Bδ from Proteintech Group (Manchester, UK; 1/400) and anti-eEF1Bγ from Abnova (Taipei, Taiwan; 1/100). The slides were then incubated and visualised using ChemMate DAKO EnVision Detection Kit (DAKO) according to the manufacturer's instructions. In brief, the slides were then washed in PBS and three drops of ChemMate DAKO Envision/HRP Rabbit/Mouse secondary antibody (DAKO Cytomation; Agilent Technologies, Wokingham, UK) were added to each slide and incubated for a further 30 minutes. The slides were washed with PBS, removed from the sequenzer and 0.5 ml of DAB working solution was added to each slide and incubated for 2 minutes. Finally, the slides were washed in dH_2_O, counterstained in haemotoxylin, stained with lithium carbonate and dehydrated in absolute ethanol and 75% ethanol, cleared in xylene and mounted in pertex. The entire procedure was performed at room temperature. Sections were viewed by light microscopy on Olympus BX51 using DP software (Olympus).

### RNA interference

Silencer siRNAs were obtained from Ambion (Paisley, UK), and experiments were controlled using untransfected cells and cells transfected with a scrambled siRNA. Two siRNAs and one Silencer Select were obtained for each eEF1B subunits. Each of the siRNAs was resuspended to a concentration of 100 µM and stored at −70°C. Transfections were performed using a Nucleofector and kits supplied by Amaxa. For each well on a 6-well plate, 0.5×10^6^ cells were pelleted, resuspended in the appropriate volume of siRNAs and 100 µl of nucleofector solution (Amaxa Biosystems, now Lonza). Cells were then subjected to nucleofection a. HeLa cells were transfected with 30 nM of either a siRNA oligonucleotide targeting a particular eEF1B subunit or a non-targeting control by nucleofection. Cells were harvested 72 h after transfection and analysed by Western blot as above.

### Cell cycle analysis

For cell cycle analysis, cells were resuspended and incubated in 500 µl propidium iodide staining solution for 20 minutes before being analysed using a Coulter EPICS XL flow cytometer (Beckman Coulter). A dot-plot was drawn of forward light scatter (FLS) against side scatter (SSC), which are influenced by size and refractive index, and all cells were gated for further analysis except dead cells and cell debris by using the EXPO ADC analysis software. A FL3 histogram with a linear x axis was used to visualise the DNA content of the cells. FL3 histogram was obtained from 10,000 events data. Multicycle AV software (Phoenix Flow systems) was used to analyse the output. Statistical comparisons were carried out by estimating the standard error of the mean and testing for significant differences from the results obtained with the non-targeting siRNA using Student's t-test.

### Cell proliferation assay

Alamar blue (Life Technologies, Paisley, UK) assays were carried out according to the manufacturer's instructions and the signal was read by fluorometry (Biotech synergy HT Plate Reader - Fisher Scientific). The fluorescence readings were taken at excitation of 560 nm and emission of 590 nm.

### Immunofluorescence

Slides were blocked for 30 minutes with donkey diluted 1∶10 in PBS. Slides were incubated with primary antibodies for 10 minutes then HRP conjugated rabbit antibody 594 and HRP conjugated sheep antibody 488, red and green respectively, were added to the slides, and the slides were incubated in the dark at room temperature for 30 minutes before being washed in PBS for 5 minutes. The slides were sealed with coverslips using Vectashield (Vector Laboratories, Peterborough, UK) hard set mounting medium with DAPI and observed under a Zeiss Axioskop 2 fluorescence microscope using appropriate filters, and pictures were captured using Smart Capture 2 software.

### Proximity Ligation Assay

PLA was performed using reagents and directions supplied in the Duolink *in situ* PLA kit, All the incubation processes were performed in a pre-warmed humidity chamber at 37°C. Negative controls included a sample with only one primary antibody to one of the target proteins, and a sample with a pair of primary antibodies raised against one target protein, and a protein that was not expected to interact with the target protein (based on function and/or subcellular localisation; TK1, antibody included with kit).
